# Intralobular pulmonary sequestration in the middle lobe supplied by a right internal mammary artery: a case report

**DOI:** 10.1186/s12890-022-02083-y

**Published:** 2022-07-26

**Authors:** Rundi Gao, Libin Jiang, Zhe Ren, Linshui Zhou

**Affiliations:** 1 The Second Clinical Medical College, Zhejiang Chinese Medical University, Hangzhou, China; 2 The First Affiliated Hospital of Zhejiang Chinese Medical University, Hangzhou, China

**Keywords:** Pulmonary sequestration, Right internal mammary artery, Multidetector computed tomography angiography

## Abstract

**Background:**

Pulmonary sequestration (PS) is a rare congenital malformation that is more common in the left lower lobe, and the thoracic aorta is the most common arterial supply.

**Case presentation:**

We describe a case of a 67-year-old man with a chief complaint of intermittent cough and hemoptysis who had been diagnosed by multidetector computed tomography angiography with right middle lobe intralobular pulmonary sequestration supplied by a right internal mammary artery. Finally, he underwent middle pulmonary lobectomy with normal postoperative recovery.

**Discussion:**

This is a rare intralobular pulmonary sequestration case for a feeding artery from the right internal mammary. Multidetector computed tomography angiography should be performed for diagnosis and preoperative evaluation once pulmonary sequestration is suspected.

## Background

Pulmonary sequestration (PS) is a rare congenital malformation characterized by a lump of nonfunctional lung tissue that receives most if not all of its arterial blood from anomalous systemic circulation arteries and that does not communicate with the tracheobronchial tree [1]. Here, we present a case of intralobular pulmonary sequestration in the right middle lobe with a systemic feeding vessel from the right internal mammary artery.

## Case presentation

**Fig. 1:**
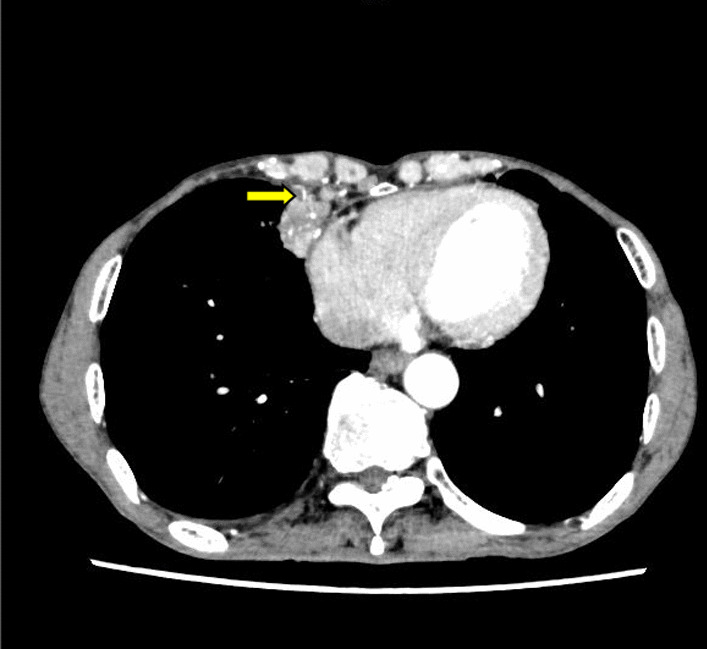
Contrast-enhanced computed tomography imaging of pulmonary sequestration. The CT scan clearly showed that the arterial supply originated from the internal thoracic artery (arrow) and extended into the mass

**Fig. 2:**
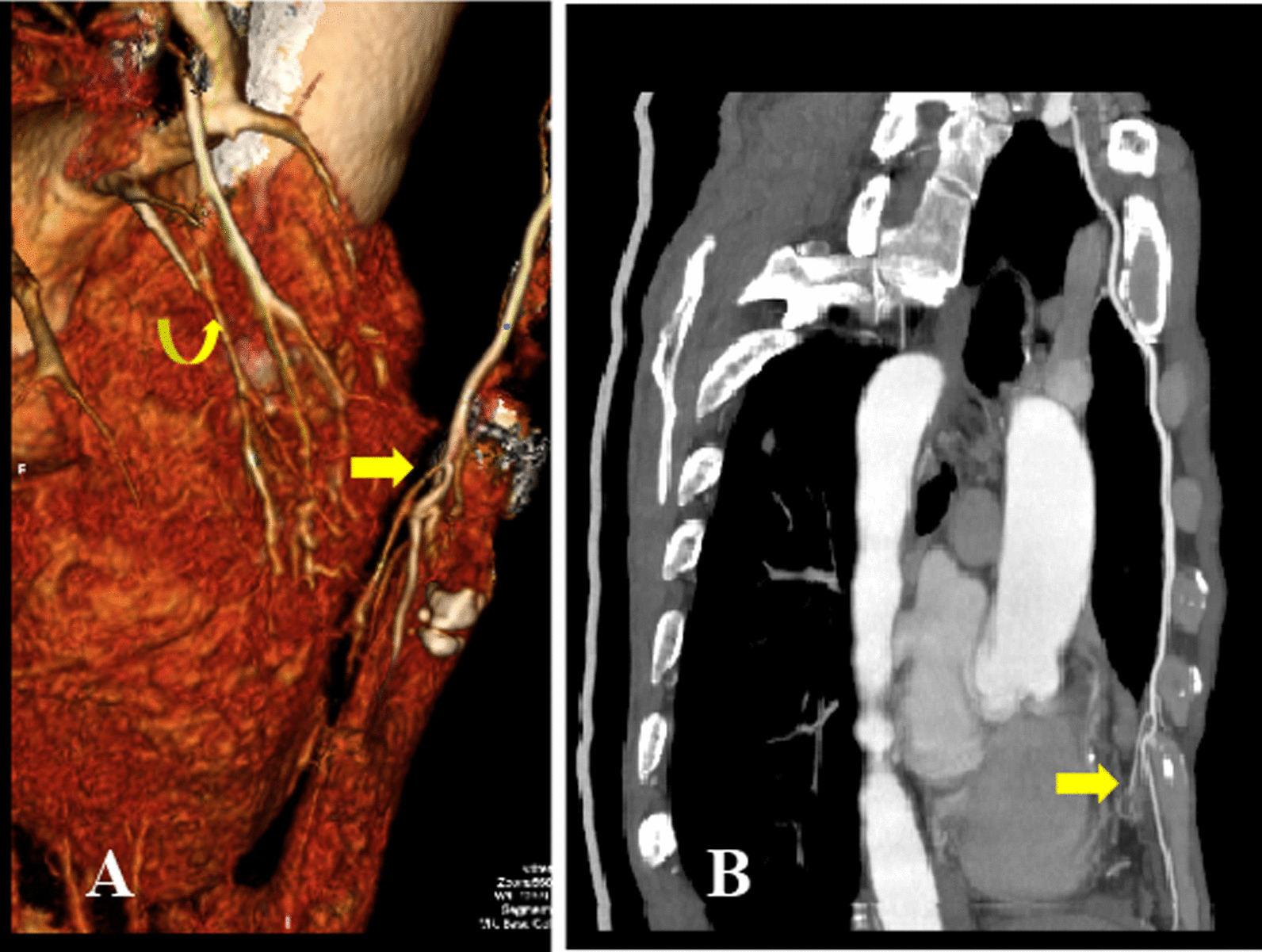
**A** 3D multidetector computed tomography volume rendering image shows an aberrant artery from the right internal mammary artery (straight arrow) and venous drainage (curved arrow) via the pulmonary veins to the right atrium. **B** Curved projection reformation (CPR) image shows the anomalous artery (straight arrow) arising from the internal mammary artery to the sequestered lung

A 67-year-old male was admitted to our hospital with intermittent cough and hemoptysis without fever. His symptoms started approximately 5 months ago with consolidation of the middle lobe of the right lung by lung computed tomography (CT). However, there was no evidence of tumor or pathogenic microorganisms by bronchoscopy. He received several courses of antibiotic for these symptoms with short-term relief. However, the lesion showed no obvious absorption on the plain CT scan. He came to our hospital for surgery because neoplastic lesions could not be excluded. On admission, his medical history and the results of his physical examination were unremarkable. Blood tests showed an increase in inflammatory markers (C-reactive protein 19.5 mg/l), while examination for tubercle bacillus and fungus was negative. Contrast-enhanced CT was performed again for preoperative evaluation. The chest CT scan showed a 36 × 23 mm consolidation mass in the medial middle lobe of the right lung, which was close to the anterior chest wall and mediastinum. In addition, obvious enhancement was observed on the contrast-enhanced scan. There were plexus vascular shadows in the mass, and a branch of the right internal mammary artery was seen crossing into the lesion. (Fig. 1). Abnormal blood supply and venous drainage were clearly seen on three-dimensional (3D) multidetector computed tomography volume rendering (VR) and curved projection reformation (CPR) images (Fig. 2). Thus, the diagnosis of intralobular pulmonary sequestration was confirmed. However, the patient was still treated by video-assisted thoracoscopic resection for right middle pulmonary lobectomy due to recurrent respiratory infection. The drainage tube was removed 4 days after surgery, and the patient was discharged one day later. At the six-month follow-up, the patient had a normal postoperative recovery without repeated pneumonia.

## Discussion

Pulmonary sequestration is a rare pulmonary malformation that was initially described by Pryce in 1946. It constitutes approximately 0.15–1.8% of all congenital pulmonary malformations, second only to cystadenoma [2]. It is usually classified into two types: intralobular sequestration, in which the malformation is located within the normal visceral pleura, and extralobular sequestration, in which the malformation is surrounded by its own pleura [3]. The most common location of the lesion is the lower lobe, and approximately two-thirds of intralobular sequestrations are found in the left lower lobe. Approximately 95% of the abnormal nutrient arteries of the PS are from the thoracic and abdominal aortas [4], and a few of them originate from other arteries, such as the intercostal artery, diaphragmatic artery, aortic arch, subclavian artery [5], left gastric artery, and coronary artery [6]. Intralobular sequestration is characterized by partial communication with the bronchi or adjacent lung tissue; it is not to be identified until complicated recurrent infection, torsion, hemoptysis, infarction, or malignant tumor have been observed [7–10] and is often confused with lung infection, bronchiectasis and lung tumor. The identification of arterial blood supply is the primary goal for the diagnosis. Previously, selective digital subtraction angiography (DSA) was used as a gold standard for characterizing pulmonary sequestration. At present, noninvasive imaging methods, including multidetector computed tomography (MDCT) angiography, color Doppler ultrasonography and magnetic resonance angiography (MRA), have gradually been regarded as suitable alternatives. In particular, MDCT angiography can be used to obtain complete chest and abdomen scans in a short time with high spatial resolution, which makes up for some limitations of MRI and ultrasound. MDCT can not only confirm the correction between the sequestered lung and systemic arterial supply but also clearly display the number, origin, course and direction of abnormal supplying arteries through 3D VR technology when combined with advanced postprocessing techniques [11]. Due to the advantages of affordable price, simple operation and high coverage provided by the equipment, this technique is widely accepted in clinical practice.

To date, there are only three cases with the internal mammary artery as the systemic feeding vessel [12–14]. Only one sequestration in the right middle lobe has been reported [13]. Digital subtraction angiography of that patient showed two anomalous systemic arteries, one from the right internal mammary artery and the other arising from the right renal artery. Here, we present a unique case of intralobular pulmonary sequestration in the right middle lobe with a single internal mammary artery supply. At first, the consolidation of inflammation in the right middle lobe had been considered a right middle lobe syndrome with suspicion of an unknown lung tumor because of the recurrent respiratory symptoms. Fortunately, multidetector computed tomography (MDCT) angiography was applied as a preoperative assessment.

The preferred treatment for symptomatic pulmonary sequestration is surgical resection via conventional thoracotomy or video-assisted thoracoscopic resection. Minimally invasive thoracoscopy is widely used because there are no differences in terms of operative duration, length of postoperative stay or complications between this and other techniques. Recently, endovascular occlusion has emerged as another surgical alternative to lobectomy, which can not only minimize intraoperative bleeding but also result in regression of the lesion [15]. Importantly, endovascular treatment alone has high recurrence rates. The difficulty of these treatments lies in the preoperative identification of the trajectory of aberrant feeding arteries, which can often be obscured in dense adhesions [16]. MDCT angiography, intuitively and clearly displaying the relationship among lung lesions, abnormal systemic suppled arteries and drainage veins of pulmonary sequestrations, is helpful for making preoperative decisions and preventing hemorrhage during surgery.

In summary, we present a rare case of intralobular pulmonary sequestration with arterial supply from the right internal mammary. MDCT angiography can accurately identify the systemic arterial supply and drainage veins of the sequestered lung as well as the location. MDCT angiography has the potential to become a first-line examination technique for the diagnosis and preoperative assessment of pulmonary sequestration.

## Data Availability

The datasets are available from the corresponding author on reasonable request.

## Abbreviations

PS: Pulmonary sequestration
CT: Computed tomography
CPR: Curved projection reformation
DSA: Digital subtraction angiography
MDCT: Multidetector computed tomography
MRA: Magnetic resonance angiography
